# Risk factors for refracture after proximal femur fragility fracture

**DOI:** 10.1097/j.pbj.0000000000000207

**Published:** 2023-04-10

**Authors:** Beatriz C. Lourenço, Tiago Amorim-Barbosa, Carolina Lemos, Ricardo Rodrigues-Pinto

**Affiliations:** a Instituto de Ciências Biomédicas Abel Salazar, Porto, Portugal; b Department of Orthopaedics, Centro Hospitalar Universitário do Porto, Porto, Portugal; c UnIGENe, i3S-Instituto de Investigação e Inovação em Saúde, Universidade do Porto, Porto, Portugal; d Department of Orthopaedics, Spinal Unit (UVM), Centro Hospitalar Universitário do Porto, Porto, Portugal

**Keywords:** fragility fracture, proximal femur fracture, refracture rate, osteoporosis

## Abstract

**Introduction:**

Proximal femur fragility fractures (PFFFs) are a growing worldwide concern. Recognizing the risk factors for subsequent fracture is essential for secondary prevention. This study aimed to analyze the risk factors for refracture and mortality rates in patients who suffered a PFFF.

**Methods:**

Patients aged 65 years or older with PFFF who underwent surgical treatment during the year of 2017 in the same institution were retrospectively analyzed and at least four years after the index fracture were evaluated.

**Results:**

From a total of 389 patients, 299 patients were included, with a median age of 83 years, and 81% female. Thirty-two (10.7%) suffered a refracture, with a mean time to refracture of 19.8 ± 14.80 months, being the female sex a risk factor for refracture (OR-4.69; CI [1.05–20.95]). The 1-year mortality rate was 15.4%. Seventy-three (24.4%) patients had previous fragility fractures. After the index fracture, 79% remained untreated for osteoporosis. No statistical association was found between antiosteoporotic treatment and refracture. Patients with refracture had higher prefracture functional level compared with patients without refracture (OR-1.33; CI [1.08–1.63]) and were discharged more often to rehabilitation units (31% versus 16%, *P* =.028). After 4 years of follow-up, patients with refracture had lower functional level compared with patients without. Chronic kidney disease was a risk factor (*P* = .029) for early refracture (<24 months).

**Conclusion:**

Female sex and higher prefracture functional level may increase the risk of refracture. Chronic kidney disease was associated with a shorter refracture time. Despite having a PFFF or other fragility fractures, the majority of patients remained untreated for osteoporosis.

## Introduction

Primary osteoporosis is a systemic chronic metabolic disease characterized by loss of bone mineral density (BMD) predisposing to an increased bone fragility and fracture.^1,2^ The most common fragility fractures are those of the hip, vertebrae, proximal humerus and distal forearm.^2^ The World Health Organization estimates that approximately one-third of the elderly fall annually, and of them, 5% will experience some type of fracture and 1% will suffer a proximal femoral fracture (PFF).^3^ These fractures are responsible for severe disability, institutionalization, reduced quality of life, and high rates of morbidity and mortality.^2,4-6^ Furthermore, their management is associated with a heavy burden to health care systems.^7,8^

The occurrence of one osteoporotic fracture is the single most predictive factor for a secondary fracture.^2,9^ Although many antiosteoporotic medications can reduce the risk of subsequent fractures, there is evidence to suggest that only a minority of patients with a fragility fracture receive treatment.^5,6^

The main aim of this study was to analyze the refracture and mortality rates in patients aged 65 years or older with proximal femur fragility fractures (PFFFs) and understand which factors influence refracture.

## Methods

Patients with PFFF who underwent surgical treatment in a single institution during the year of 2017 were retrospectively analyzed. Data from the two years prior and from the four years after the index fracture (IF) were analyzed. The study was approved by the local ethics committee.

### Data collection

Patient's information was collected by using clinical records and telephone interview. The analysis of electronical clinical files allowed to collect information for the following variables: sex and age at the time of the IF, mortality, Charlson Comorbidity Index (CCI) at the time of the IF, number and location of fragility fractures before and subsequent to the IF, delivery of antiosteoporotic medication in the 2 years prior and after the IF, type of primary fracture (femoral neck, trochanteric and subtrochanteric fractures), American Society of Anaesthesiologists (ASA) grade, preoperative hemoglobin and need for transfusion, type of surgical procedure for IF (antegrade nailing, sliding hip screw, hemiarthroplasty, and total hip arthroplasty), duration of surgery, and length of hospital stay (LHS).

The telephone survey included the following variables: patient's orientation after hospital discharge (home, nursing home, or rehabilitation units), prescription, and compliance with the antiosteoporotic medication in the 2 years prior and after the IF and patient's mobility at last follow-up. Antiosteoporotic treatment was considered complete when patients were under the administration of an antiosteoporotic agent with proven effectiveness in reducing the incidence of fragility fractures (bisphosphonates, calcitonin, strontium ranelate, parathormone, or denosumab) with or without calcium/vitamin D supplements in the 2 years preceding the IF and/or during the follow-up.

CCI predicts the 10-year mortality for a patient with various comorbidities that are weighted from 1 to 6 for mortality risk and disease severity and then summed to form the total CCI.^10^ New Mobility Score (NMS) is considered to be the most consistent predictor of rehabilitation outcome in PFF in the elderly patients.^11^ To assess patient's mobility before and at 4 years after the IF, this score was applied during the telephone interview, as has been previously done before by others.^12,13^

Patients were divided into two groups: group A (GA) consisted of patients who did not suffer a contralateral PFF during the follow-up and group B (GB) consisted of patients who suffered a contralateral PFF during the follow-up. In GA, patients with previous PFF were excluded. Age at the time of contralateral hip fracture and time to refracture, that was defined as time from IF to subsequent hospitalization for osteoporotic fracture of the contralateral proximal femur, was analyzed in GB. According to refracture time, GB was divided into two subgroups: GB1 corresponds to patients with a refracture in the first 24 months and GB2 to patients with a refracture ≥24 months after IF.

### Statistical analysis

Descriptive statistics was expressed in frequencies and percentages for categorical variables and mean with standard variation (SD) for continuous variables. Categorical variables were assessed using Chi-Square test or Fisher exact test as appropriated. For continuous variables, a Student *t* test for independent variables was used, after normal distribution confirmation. To compare NMS level before and after the IF, a paired samples *t* test was used. Variables were considered statistically significant for a *P* value <.05. Statistical analyses were performed with SPSS 27.0. A logistic regression was also performed, and the odds ratio (OR) and confidence intervals (CI) were also estimated.

## Results

From a total of 389 patients with PFFF who underwent surgical treatment in the same institution, 299 patients were included. Ninety patients were excluded: 2 patients with pathological fractures, 3 suffered high-energy trauma, 32 younger than 65 years, 4 had periprosthetic hip fractures, and 49 did not respond or refused to participate in the study.

Sociodemographic and clinical characteristics are shown in Table 1. GA had 238 patients (79.6%) and GB had 32 patients (10.7%), with 21 patients (65.6%) in GB1 and 11 patients (34.4%) in GB2. The median age at the IF was 83 ± 7 years with a female predominance (n = 242, 80.90%) (*P* = .044). Between GA and GB, no difference was found regarding age at the IF (83 ± 7.7 years and 82.8 ± 8.1 years, respectively) (*P* = .869). Regarding sex, most patients were female in GA (77.7%) and GB (93.8%); however, the proportion of men was higher in the first group (53 patients, 22.3%) compared with GB (2 patients, 6.3%) (*P* = .035). The odds of having a contralateral PFF during the follow-up were 4.65 times higher in women (OR 4.69; CI [1.05–20.95]; *P* = .043) (Table 2).

**Table 1:** Demographic and clinical features of patients with and without contralateral proximal femoral fracture

	Patients without contralateral PFFF (A),n = 238 (79.6%)		Patients with contralateral PFFF (B), n = 32 (10.7%)		*P*
	n (%)	Mean ± SD	n (%)	Mean ± SD	
Sex, n (%)					0.035
Female	185 (77.7)		30 (93.8)		
Male	53 (22.3)		2 (6.3)		
Age (years)		82.99 ± 7.73		82.75 ± 8.12	0.869
Deaths, n (%)	122 (51.3)		12 (37.5)		0.144
First 12 months	39 (32.0)		1 (8.3)		0.108
After 12 months	83 (68.0)		11 (91.7)		
Hospital stay (days)		17.24 ± 14.71		20.41 ± 13.38	0.249
ASA					
1	82 (34.4)		12 (37.5)		
2	137 (57.6)		16 (50.0)		
3	18 (7.6)		4 (12.5)		
4	1 (0.4)		0 (0.0)		
Charlson Comorbidity Index, median ±SD		6.13 ± 2.00		5.72 ± 1.91	0.279
Stroke or TIA	46(19.3)		5(15.6)		0.615
Dementia	89(37.4)		10(31.3)		0.498
Hemiplegia	15(6.3)		0(0.0)		0.229
COPD	21(8.8)		2(6.3)		1.000
Myocardial infarction	36(15.1)		3(9.4)		0.591
Heart failure	91(38.2)		14(43.8)		0.548
Diabetes mellitus	66(27.7)		5(15.6)		0.144
Connective tissue disease	5(2.1)		0(0.0)		1.000
Liver disease					
Chronic kidney disease	5(2.1)		0(0.0)		1.000
Peptic ulcer disease	40(16.8)		8(25.0)		0.255
Peripheral vascular disease	6(2.5)		1(3.1)		0.591
Disease	13(5.5)		2(6.3)		0.694
NMS before index fracture		6.34 ± 2.82		8.06 ± 1.65	<0.001
Inside the house		2.61 ± 0.73		2.91 ± 0.30	<0.001
Outside the house		2.17 ± 1.07		2.72 ± 0.58	<0.001
Shopping		1.58 ± 1.49		2.44 ± 1.01	<0.001
NMS after 4 years of follow-up		4.15 ± 3.40		1.95 ± 2.01	<0.001
Inside the house		1.92 ± 1.23		1.30 ± 1.08	0.036
Outside the house		1.35 ± 1.28		0.55 ± 0.89	0.001
Shopping (n = 116 for group A; n = 20 for group B)		0.87 ± 1.28		0.10 ± 0.45	<0.001
Destination after hospital discharge	(n = 220)		(n = 32)		0.028
Home or nursing home	186 (84.5)		22 (68.8)		
RNCCI	34 (15.5)		10 (31.3)		
Other fragility fractures before index fracture					
Yes	37 (15.5)		7 (21.9)		0.363
No	201 (84.5)		25 (78.1)		
Antiosteoporotic medication during the follow-up period					
Yes	48 (21.8)		5 (15.6)		0.422
No	172 (78.2)		27 (84.4)		

**Table 2:** Analysis of predictors of refracture of proximal femur.

Variable	*P*	OR (95% CI)
Sex (women vs. men)	.043	4.69 (1.05–20.95)
NMS score before index fracture	.006	1.33 (1.08–1.63)
Destination after hospital discharge (RNCCI vs. home/nursing house)	.035	2.55 (1.07–6.09)

CI, 95% confidence interval; OR, odds ratio.

The mean time to refracture was 19.78 ± 14.80 months, and the mean age at the time of the refracture of the proximal femur was 84.1 ± 1.5 years. GB had also a higher proportion (21.9%) of fragility fractures before the IF, compared with GA (15.5%), but without statistical significance (*P* = .363).

From the 47 patients who suffered fragility fractures during the follow-up, 15 patients (5.0%) suffered other fragility fractures than proximal femur, in which 7 were of the proximal humerus (2.5%), 4 of the distal forearm (1.4%), 3 were vertebral (1.1%), and 1 was of the ankle (0.4%).

### Mortality

A total of 151 patients (50.5%) died during the follow-up. The 1-year mortality rate was 15.4% (46 patients), of whom 20 patients (6.7%) died during the hospital stay and 2 patients (0.7%) died during the first 30 days after surgery (Table 1). In GA, 122 patients (51.3%) and 12 patients (37.5%) of GB died during the follow-up (*P* = .144).

### Functional level and CCI

Before the IF follow-up, functional level was higher (8.06 ± 1.65) in GB, compared with GA (6.34 ± 2.82) (*P* < .001) (Table 1). The odds of having a contralateral PFF were 1.33 times higher with increasing NMS before IF (OR 1.33; CI [1.08–1.63]; *P* = .006) (Table 2). After 4 years of follow-up, the mean value of NMS in GB was lower (1.95 ± 2.01) compared with GA (4.15 ± 3.40) (*P* < .001) (Table 1).

The CCI was calculated at the moment of the IF, and the mean value was 6.07 ± 2.00. Cardiac heart failure (38.8%), dementia (37.8%), diabetes (27.5%), history of stroke or transient ischemic attack (TIA) (19.7%), and CKD (17.4%) were the most frequent comorbidities. Analysis of CCI showed no statistically significant difference between groups A/B, with a mean value of 6.13 ± 2.00 and 5.72 ± 1.91, respectively. When comparing GB1 with GB2 (Table 3), the analysis showed that the first group had a higher prefracture CCI score (*P* = .031) (6.24 ± 1.92 versus 4.73 ± 1.45). It was found that the 8 patients with CKD suffered refracture in the first 24 months (*P* = .029).

**Table 3:** Time to contralateral proximal femoral fracture.

Variable	Time to contralateral proximal femoral fracture (months) <24 months (B1), n = 21, 65.6%		Time to contralateral proximal femoral fracture (months) ≥24 months (B2), n = 11, 34.4%		*P*
	n (%)	Mean ± SD	n (%)	Mean ± SD	
Sex, n (%)					
Female	19 (90.5)		11 (100.0)		0.534
Male	2 (9.5)		0(0.0)		
Age at admission of index fracture, years		82.81 ± 7.86		82.64 ± 8.99	0.955
Age at time of refracture, years		83.24 ± 8.28		85.73 ± 9.11	0.441
Charlson Comorbidity Index, median ±SD		6.24 ± 1.92		4.73 ± 1.45	0.031
Stroke or TIA	4(19.0)		5(15.6)		0.474
Dementia	8 (38.1)		2 (18.2)		0.425
Hemiplegia	0 (0.0)		0 (0.0)		0.231
COPD	1 (4.8)		1 (9.1)		1.000
Myocardial infarction	3 (14.3)		0 (0.0)		0.534
Heart failure	12 (57.1)		2 (18.2)		0.111
Diabetes mellitus	4 (19.0)		1 (9.1)		0.637
Chronic kidney disease	8 (38.1)		0 (0.0)		0.029
Deaths, n (%)	11 (52.4)		1 (9.1)		0.023
First 12 months	1 (9.1)		0 (0.0)		
After 12 months	10 (90.9)		1 (100.0)		1.000
Delivery of antiosteoporotic medication in the 2 years before index fracture, n (%)					
Yes	5 (23.8)		3 (27.3)		0.667
No	16 (76.0)		8 (72.7)		
Delivery of antiosteoporotic medication during the follow-up period, n (%)					
Yes	3 (14.3)		2 (18.2)		1.000
No	18 (85.7)		9 (81.8)		
Destination after hospital discharge, n (%)					
Home or nursing home	12 (57.1)		10 (90.9)		0.106
RNCCI	9 (42.9)		1 (9.1)		
NMS before index fracture		8.38 ± 1.43		7.45 ± 1.92	0.132
NMS after 4 years of follow-up (n = 10 for group B1 and B2)		1.80 ± 1.48		2.10 ± 2.51	0.750

### Destination after hospital discharge

A total of 186 patients (66.7%) returned to their own homes while 47 (16.8%) were discharged to rehabilitation units and 46 (16.5%) were discharged to nursing homes permanently. Patients who were discharged to rehabilitation units had higher prefracture functional level (7.25 ± 2.27) compared with patients who returned home or to a nursing home (6.50 ± 2.81) (*P* = .048). The results showed that the proportion of patients who were discharged to rehabilitation units was higher in GB (31.3%) compared with GA (15.5%) (*P* = .028) (Table 1). Patients who were discharged to rehabilitation units were 2.55 times more likely to have contralateral PFF compared with patients who returned home or to a nursing home (OR 2.55; CI [1.07–6.09]; *P* = .035) (Table 2).

### Antiosteoporotic treatment

In the 2 years preceding the IF, 252 patients (84.3%) did not receive any medication and 47 patients (15.7%) had received antiosteoporotic treatment. Twenty-five patients (8.4%) were using calcium and/or vitamin D supplements, and 21 patients (7%) were under complete osteoporosis treatment. A large difference in treatment rates was observed, with 91.5% of women receiving treatment before the IF, compared with 8.5% of men (*P* = .045). After the IF, 220 patients (78.9%) remained without osteoporosis treatment and 59 (21.1%) patients received antiosteoporotic treatment. Of these patients, most (13.6%) were treated using calcium and/or vitamin D supplements alone and only 14 patients (5%) were under complete osteoporosis treatment. GB had a higher proportion of patients (21.9%) who were under treatment before the IF compared with 13.9% from GA, but without statistically significant difference (*P* = .285). After the IF, the proportion of patients receiving treatment was lower in GB (15.6%) compared with GA (21.8%), but not statistically significant (*P* = .422). The proportion of patients with previous PFFF who had received antiosteoporotic medication in the 2 years before IF was similar (n = 6, 22.2%) to those without previous fragility fractures (n = 34 patients, 15%) (*P* = .279) (Table 4).

**Table 4:** History of previous fragility fractures.

Variable	Without previous fragility fractures, n = 226, 75.6%		With a previous proximal femoral fracture, n = 29, 9.7%		*P*
	n (%)	Mean ± SD	n (%)	Mean ± SD	
Sex, n (%)					
Female	176 (77.9)		27 (93.1)		0.055
Male	50 (22.1)		2 (6.9)		
Age at admission of index fracture, years		82.68 ± 7.96		84.10 ± 8.93	0.371
Deaths, n (%)	111(49.1)		17 (58.6)		0.335
First month	18 (52.9)		3 (50.0)		1.000
First 12 months	16 (47.1)		3 (50.0)		
Delivery of antiosteoporotic medication in the 2 years before index fracture, n (%)					
Yes	34 (15.0)		7 (24.1)		0.279
No	192 (85.0)		22 (75.9)		
Delivery of antiosteoporotic medication during the follow-up period, n (%)					
Yes	46 (22.0)		6 (22.2)		0.980
No	163 (78.0)		21 (77.8)		
Destination after hospital discharge, n (%)					
Home or nursing home	170 (81.8)		23 (85.2)		0.794
RNCCI	39 (18.2)		4 (14.8)		
NMS before index fracture		6.59 ± 2.69		5.79 ± 2.72	0.133
NMS after 4 years of follow-up		3.97 ± 3.27		2.67 ± 1.92	0.053
Charlson Comorbidity Index, median ±SD		6.05 ± 1.97		6.03 ± 2.13	0.971

### Previous fragility fractures

In 226 patients (75.6%), the index PFF was the first presentation of a fragility fracture, and 73 (24.4%) patients had sustained additional fragility fractures before the IF. The most common sites of previous fragility fractures included the distal forearm (n = 19, 6.4%), the proximal humerus (n = 17, 5.7%), and the spine (n = 4, 1.3%). Of those 73 patients, 29 (9.7%) had contralateral PFF before the IF, of whom 27 (93.1%) were females and 2 (6.9%) males.

### IF and surgical procedure-related variable

Most of the PFF were extracapsular (178 patients (59.5%) with a trochanteric and 18 patients (6.0%) with a subtrochanteric fracture) over intracapsular fractures (103 patients, 34.4%) (Table 5). Most patients (55.5%) were ASA 2. The mean value of preoperative hemoglobin was 11.90 ± 1.51 mg/dL, and 69 patients (23.1%) needed transfusion. The mean LHS was 17.38 ± 14.21 days. Patients who suffered femoral neck fracture were found to be younger (81.61 ± 7.41 years) compared with those who suffered trochanteric or subtrochanteric fractures (83.84 ± 8.02 years) (*P* = .020).

**Table 5:** Index fracture classification.

Variable	Femoral neck, n = 103, 34.4%		Trochanteric and subtrochanteric, n = 196, 65.6%		*P*
	n (%)	Mean ± SD	n (%)	Mean ± SD	
Age at index fracture, years		81.61 ± 7.41		83.84 ± 8.02	0.020
Sex, n (%)					
Female	84 (81.6)		158 (80.6)		0.844
Male	19 (18.4)		38 (19.4)		
Deaths, n (%)					
First 30 days after surgery	9 (42.9)		13 (52.0)		0.536
Between 1 and 12 months	12 (57.1)		12 (48.0)		
ASA					
1	29 (28.2)		76 (38.8)		
2	61 (59.2)		105 (53.6)		
3	12 (11.7)		15 (7.7)		
4	1 (1.0)		0 (0.0)		
Surgical procedure, n (%)					
Antegrade nailing and Sliding hip screw	36 (35.3)		191 (97.4)		
Hemiarthroplasty and total hip arthroplasty	66 (64.7)		5 (2.6)		<0.001
Duration of the surgery, minutes		91.73 ± 37.56		63.99 ± 27.62	<0.001
Length of hospital stay, days		16.50 ± 14.29		17.84 ± 14.18	0.437
Need for transfusion, n (%)					
Yes	21 (20.4)		48 (24.5)		0.424
No	82 (79.6)		148 (75.5)		
Destination after hospital discharge, n (%)					
Home or nursing home	82 (86.3)		149 (81.0)		0.263
RNCCI	13 (13.7)		35 (19.0)		
NMS before index fracture		6.11 ± 2.93		6.66 ± 2.66	0.098
NMS after 4 years of follow-up		3.85 ± 2.85		3.67 ± 3.44	0.735

Regarding surgical procedure, 175 patients (58.5%) underwent antegrade nailing, 66 (22.1%) total hip arthroplasty, 52 (17.4%) sliding hip screw, 5 (1.7%) hemiarthroplasty, and 1 (0.3%) resection arthroplasty. Regarding time from fracture to surgery (Table 6), the mean value was 2.98 ± 2.35 days, with 128 patients (42.8%) submitted to surgery in the first 48 hours and 171 patients (57.2%) after 48 hours. Patients who underwent surgery in the first 48 hours had a lower LHS (13.50 ± 11.02 compared with 20.28 ± 15.61) (*P* < .001). No difference was found regarding mortality rate between these two groups (*P* = .933).

**Table 6:** Time from fracture to surgery.

Variable	Time from fracture to surgery <48 hours, n = 128 (42.8%)		Time from fracture to surgery ≥48 hours, n = 171 (57.2%)		*P*
	n (%)	Mean ± SD	n (%)	Mean ± SD	
Deaths, n (%)	65 (50.8)		86 (50.3)		0.933
First 30 days	18 (27.7)		28 (32.6)		
Between 1 and 12 months	47 (72.3)		58 (67.4)		0.520
Length of hospital stay, days		13.50 ± 11.02		20.28 ± 15.61	<0.001
Destination after hospital discharge, n (%)					
Home or nursing home	104 (86.7)		127 (79.9)		0.137
RNCCI	16 (13.3)		32 (20.1)		
NMS before index fracture		6.55 ± 2.76		6.42 ± 2.77	0.684
NMS after 4 years of follow-up		3.48 ± 3.47		3.92 ± 3.06	0.414
Charlson Comorbidity Index, median ±SD		6.06 ± 2.05		6.08 ± 1.98	0.934

## Discussion

The results of this study demonstrate that having a PFF was associated with increased mortality. After the IF, 50.5% of patients died during the follow-up, with 15.4% of the deaths occurring in the first year and 6.7% of deaths occurring during hospital stay. Similar rates were found in the studies by Roux,^7^ with a mortality rate at 1 year of 16.6% and with 3.4% of deaths during hospitalization. The estimated expected yearly mortality for the general population, of similar age and sex composition, is approximately 8.6%.^8^ The mortality rate was 37.5% in GB versus 51.3% in GA. However, these results are difficult to interpret because of sample size discrepancy between the two groups. Other studies reported increased mortality in patients with higher CCI, higher degree of dependence, male sex and aged older than 80 years.^14^

The incidence of contralateral PFF was 10.7%. This is consistent with what has been found in previous studies.^6,15-17^ The mean time to refracture was 2.5 years, which is considered the high-risk period for refracture of the proximal femur after a previous PFF. The time to refracture in the literature ranges from 3 to 67 months.^6,15-18^ The mean age of the 32 patients who suffered a contralateral PFF at the time of the IF fracture was 82.75 ± 8.12 years and 93.8% were women, and this finding is consistent with what has been described.^17^

Most patients with a PFF were female (80.9%), and this difference was even more pronounced in those who suffered contralateral PFF (93.8%), which is in accordance with some authors.^6,8,15,19^ However, when analyzing the patients who suffered refracture, there is an important sample size discrepancy regarding sex, which can explain the low precision (CI [1.05–20.95]) and the more pronounced female prevalence in this group. Therefore, female sex was found to be predictive of refracture with 4.69 more risk for refracture of proximal femur. A previous study by Alpantaki^20^ reported an increased risk in women but lower than in the current report, with women suffering hip fractures 2.9 times more often than men. This can be justified by estrogenic deficiency in postmenopausal women which leads to loss of trabecular bone.^3^ Also notable in the current study is that women not only have a higher risk of refracture but also a higher risk of having other fragility fractures. Crandall^19^ reported that, in postmenopausal women, any type of initial fragility fracture was associated with an increased risk of subsequent fracture. Regarding sex, only two men sustained a contralateral PFF, and it occurred in the first 24 months. Although these findings showed no statistically significant differences regarding male sex, it must be noted that other studies found that men have a shorter refracture time in cases of contralateral hip fracture.^6^

Higher prefracture functional level was considered predictive of refracture in this study. Although this finding was somewhat surprising, a similar finding was reached by Bosco,^6^ who demonstrated that a NMS ≥5 between the IF and the refracture was associated with a greater risk of contralateral PFF and a shorter refracture time. This suggests that higher mobility is associated with a higher risk of falls and, consequently, fracture of the proximal femur. Four years after the IF, there was a marked decrease in functional capacity. In fact, patients who suffered a contralateral PFF had a significantly lower functional level than patients who sustained only the IF. This represents the negative impact of suffering two PFF in their physical functioning and quality of life. Therefore, rehabilitation programs and measures to prevent falls after fracture should begin as soon as possible to restore the patient's prefracture activity status and reduce the risk of falls.^14,21^

This study confirmed that the most frequent comorbidities associated with PFF fractures are heart failure, dementia, diabetes, and history of stroke or TIA and CKD. Heart failure and CKD were more prevalent in patients who suffered refracture, but this difference failed to reach statistical significance. Carbone^22^ concluded that patients with heart failure are at high risk for hip fractures not only because both of their cumulative high prevalence in the elderly population but also because they share various risk factors. Dementia and having history of stroke or TIA, because of cognitive impairment and disability, are associated with as increased risk of falling.^23,24^

The results also demonstrated that patients with CKD had a shorter refracture time. Various studies have reported that the hip fracture risk is 4–13 times higher in patients with end-stage kidney disease.^25-28^ In fact, mineral and bone disease (MBD) observed in CKD are now a whole entity called CKD-MBD, which refers to clinical events related to secondary hyperparathyroidism due to phosphorus accumulation in circulation, leading to an increase in the risk of cardiovascular disease and fracture.^29-31^ Uremia, a consequence of renal insufficiency, is likely to deteriorate bone material properties and cause cognitive dysfunction which also increases the risk of falls.^30^ Innumerous factors, such as abnormal vitamin D metabolism, hypoproteinemia, secondary hyperparathyroidism, hypocalcemia and bone metabolic abnormalities, are considered causative factors for decreased bone mass in these patients. Previous studies have shown that COPD is a risk factor for contralateral PFF, because of muscle wasting, smoking, low BMD, or inhaled or systemic corticosteroid use. However, this study did not show a correlation between COPD and the risk of refracture and refracture timing.^14,24,32,33^

Being discharged to rehabilitation units after the IF was also considered a predictor of refracture, with a 2.55 increased risk associated. However, it may be considered a confounding factor because patients who were discharged to rehabilitation units were found to have higher NMS, which was associated with increased risk of refracture. Furthermore, national rehabilitation facilities are often used for patients with lack of social support and not particularly to provide intensive rehabilitation and, therefore, may not necessarily serve as surrogates for appropriate rehabilitation.

Regarding bone protection before the IF, 7% of patients were under complete osteoporosis treatment, which was lower compared with other studies. Aguado-Maestro^4^ and Roux^7^ respectively reported a total of 16.4% and 17% patients who were under complete osteoporosis in the 2 years before the IF. After the IF event, only 5% of the patients received and completed complete osteoporosis treatment, in spite of the fact that antiosteoporotic medication can control bone loss and reduce the risk of refracture.^5,6,15,16,34^ This percentage was lower compared with the findings by Roux^7^ who reported a total of 20.7% patients under complete treatment. In this study, suffering a refracture did not increase the proportion of patients under treatment. In those who suffered refracture, the group with a time to refracture <24 months had a lower proportion of patients (9.4%) under treatment compared with 18.2% patients with a time to refracture ≥24 months. However, these differences failed to reach statistical significance. Therefore, the results of this study do not allow us to conclude that antiosteoporotic treatment reduces the risk of refracture of the contralateral proximal femur, which is in agreement with the findings in the study by Bosco.^6^

Among those who were taking osteoporosis medication, higher treatment rates were found in women, which is consistent with previous studies.^35^ This finding can be explained by the fact that osteoporosis is more common in women, and postmenopausal women are at a higher risk of fractures with twice the increased risk in comparison with men because of estrogen decline during menopause.^2,36^

The large proportion of patients at high risk of fractures who remain untreated after the IF occurs mainly due to two reasons: no prescription or patients not adhering to treatment.^37^ Oral bisphosphonates, despite being an effective antiosteoporotic agent, require patient to stand or to sit up straight for 30 minutes, which can be challenging for elderly patients who are not able to walk without difficulty. Other reason that few studies reported as reason for nonadherence to the medication is because of fear of rare side effects and concerns regarding the long-term efficacy of these treatment.^37^ For that reason, according to national guidelines, clinicians must identify patients at high risk of fragility fractures and to start antiosteoporotic treatment and prescribe effective antiosteoporotic agents according to the patient's comorbidities and investigate adherence to therapy, despite correctly instituted therapy.^38^

When considering previous fragility fractures, we found that prevalence was lower comparing the literature (24.4% versus [30.7–45] %).^4,34,39^ This study found that patients who suffered a contralateral PFF during the follow-up also had a higher proportion (21.9%) of other fragility fractures before the IF compared with those who did not suffer refracture (16.4%). According to national guidelines, fractures in locations other than the proximal femur are not only considered to be diagnostic of osteoporosis but also justify the initiation of antiosteoporotic treatment, which was absent in 82.2% of high-risk patients (those with previous fragility fractures). Even after the IF, 81.4% of these high-risk patients with two or more fragility fractures were not taking any type of antiosteoporotic medication. From this standpoint, despite the high prevalence of fragility fractures, the benefits of antiosteoporotic treatment in improving bone strength and cost-effective reduction in the risk of subsequent fragility fractures, and the recommendations to start antiosteoporotic treatment, a large proportion of patients remain untreated.^40^ Furthermore, because 66.5% of contralateral PFF occurred after 2 years, a large window of opportunity exists to initiate medical treatment for osteoporosis to reduce the rate of refracture, to save costs, and to decrease morbidity and mortality.

Contrary to the finding of Ganhão^14^ who reported that femoral neck fracture was associated with increased mortality, we did not find difference between femoral neck and trochanteric/subtrochanteric fractures. In fact, most of this type of fractures underwent total arthroplasty or hemiarthroplasty which is a more complex surgery associated with more postoperative complications. Extracapsular fractures are more strongly associated with advanced age and osteoporosis.^2,20^ For this reason, older patients who are more likely to have advanced osteoporosis tend to suffer more frequently from extracapsular fractures, which can be seen in the results of this study. To achieve best outcomes, evidence supports that hip fracture surgery should take place within 48 hours after admission and, ideally, within the first 24 hours.^21,41,42^ To achieve this goal, these patients should be evaluated by an interdisciplinary team (including orthopaedics, internal medicine/family medicine, anesthesiology, and nursing). Early surgery reduces complications, pain, and LHS.^43^ In this study, 57.2% of patients underwent surgery after 48 hours. This reason can also explain the longer LHS in patients who were submitted to surgery after 48 hours. However, and contrary to Leer-Salvesen,^44^ delayed surgery was not associated with increased mortality.

This study has some limitations. The first limitation is that the design of the study was retrospective. The analysis of functional level was assessed by telephone interview of patients or their family members using the NMS. How the information may not be as reliable as if an objective assessment of physical performance had been carried out. However, this score has previously been assessed in the same manner in other studies.^12,13^ In those who died, the source of information was a family member. Another limitation is the absence of an intermediate NMS level at 1-year postoperatively. The results showed that a higher prefracture NMS level was associated with an increased risk of refracture. However, the study does not provide information about whether patients with refracture were more autonomous after the IF than those who did not suffer a contralateral fracture at that time.

## Conclusion

Female sex and higher prefracture functional level are associated with higher risk of refracture. CKD were associated with a shorter refracture time. Despite having a PFFF and prior fragility fractures, most patients remain untreated for osteoporosis. Although antiosteoporotic treatment was not a protective factor for refracture in this study, given the high mortality rate related to PFF and mobility problems, their occurrence must be identified as a warning sign that improves patients' risk of potential fragility fractures. It is also an opportunity to initiate osteoporosis medical treatment and to optimize their rehabilitation outcome.

## Conflict of interest

The authors declare no conflict of interests.
